# Detection of Intramyocardial Iron in Patients Following ST‐Elevation Myocardial Infarction Using Cardiac Diffusion Tensor Imaging

**DOI:** 10.1002/jmri.28063

**Published:** 2022-01-12

**Authors:** Arka Das, Christopher Kelly, Irvin Teh, Noor Sharrack, Christian T. Stoeck, Sebastian Kozerke, Jürgen E. Schneider, Sven Plein, Erica Dall'Armellina

**Affiliations:** ^1^ Biomedical Imaging Science Department Leeds Institute of Cardiovascular and Metabolic Medicine, University of Leeds, Leeds Teaching Hospitals NHS Trust Leeds UK; ^2^ Institute for Biomedical Engineering, University and ETH Zurich Zurich Switzerland

**Keywords:** cardiac magnetic resonance, diffusion tensor imaging, myocardial infarction, intramyocardial hemorrhage

## Abstract

**Background:**

Intramyocardial hemorrhage (IMH) following ST‐elevation myocardial infarction (STEMI) is associated with poor prognosis. In cardiac magnetic resonance (MR), T2* mapping is the reference standard for detecting IMH while cardiac diffusion tensor imaging (cDTI) can characterize myocardial architecture via fractional anisotropy (FA) and mean diffusivity (MD) of water molecules. The value of cDTI in the detection of IMH is not currently known.

**Hypothesis:**

cDTI can detect IMH post‐STEMI.

**Study Type:**

Prospective.

**Subjects:**

A total of 50 patients (20% female) scanned at 1‐week (V1) and 3‐month (V2) post‐STEMI.

**Field Strength/Sequence:**

A 3.0 T; inversion‐recovery T1‐weighted‐imaging, multigradient‐echo T2* mapping, spin‐echo cDTI.

**Assessment:**

T2* maps were analyzed to detect IMH (defined as areas with T2* < 20 msec within areas of infarction). cDTI images were co‐registered to produce averaged diffusion‐weighted‐images (DWIs), MD, and FA maps; hypointense areas were manually planimetered for IMH quantification.

**Statistics:**

On averaged DWI, the presence of hypointense signal in areas matching IMH on T2* maps constituted to true‐positive detection of iron. Independent samples *t*‐tests were used to compare regional cDTI values. Results were considered statistically significant at *P* ≤ 0.05.

**Results:**

At V1, 24 patients had IMH on T2*. On averaged DWI, all 24 patients had hypointense signal in matching areas. IMH size derived using averaged‐DWI was nonsignificantly greater than from T2* (2.0 ± 1.0 cm^2^ vs 1.89 ± 0.96 cm^2^, *P* = 0.69). Compared to surrounding infarcted myocardium, MD was significantly reduced (1.29 ± 0.20 × 10^−3^ mm^2^/sec vs 1.75 ± 0.16 × 10^−3^ mm^2^/sec) and FA was significantly increased (0.40 ± 0.07 vs 0.23 ± 0.03) within areas of IMH. By V2, all 24 patients with acute IMH continued to have hypointense signals on averaged‐DWI in the affected area. T2* detected IMH in 96% of these patients. Overall, averaged‐DWI had 100% sensitivity and 96% specificity for the detection of IMH.

**Data Conclusion:**

This study demonstrates that the parameters MD and FA are susceptible to the paramagnetic properties of iron, enabling cDTI to detect IMH.

**Evidence Level:**

1

**Technical Efficacy:**

Stage 2

Advancements in primary percutaneous coronary intervention (PPCI) have improved outcomes following ST‐elevation myocardial infarction (STEMI); however, microvascular obstruction (MVO) as detected by cardiac magnetic resonance (MR), occurs in approximately 50% of patients following reperfusion and is associated with worse prognosis.^1^ Reperfusion of severely ischemic myocardium can lead to intramyocardial hemorrhage (IMH) within areas of MVO by extravasation of red blood cells through damaged endothelial walls. The paramagnetic properties and magnetic susceptibility effects of hemosiderin—a breakdown product of red blood cells—can shorten T2 and T2* relaxation times on MR imaging.^2^ On T2‐weighted (T2W) imaging, IMH is typically characterized as hypointense regions surrounded by edema (bright signal), a finding that has been corroborated against histology.^3^ On T2* mapping, regions with relaxation times <20 msec indicate the presence of iron.^4^ Acute IMH and the presence of residual iron in chronic infarct segments following STEMI are associated with increased risk of adverse LV remodeling and ventricular arrhythmia.^2^, ^5^

Cardiac diffusion tensor imaging (cDTI) is sensitive to the random motion of water molecules and can measure the mean diffusivity (MD) and the fractional anisotropy (FA) of diffusion within the myocardium. These measurements allow characterization of edema and scar, as well as detecting irreversible injury to microstructural components following STEMI.^6^, ^7^ cDTI is intrinsically T2 weighted, thus the obtained images are also susceptible to the paramagnetic properties of hemosiderin. In diffusion‐weighted imaging (DWI) of the brain, previous studies have shown iron deposition to have a substantial impact on regional MD and FA values.^8^

In the present study, we hypothesized that cDTI could also depict areas of IMH and residual iron in the heart following STEMI.

## Methods

### Patient Recruitment

The study protocol was approved by the institutional research ethics committee and complied with the Declaration of Helsinki (NIHR 33963, REC 17/YH/0062). Patients with a first STEMI were prospectively recruited from a single center between 2019 and 2020 and underwent serial cardiac MR at 1 week and 3 months following their index presentation. Study inclusion criteria were 1) acute STEMI as defined by current international guidelines,^9^ 2) revascularization via PPCI within 12 hours after onset of symptoms, and 3) no contraindications to cardiac MR. Exclusion criteria were 1) previous revascularization procedure (coronary artery bypass grafting or PPCI), 2) known cardiomyopathy, 3) severe valvular heart disease, 4) atrial fibrillation, and 5) hemodynamic instability lasting longer than 24 hours following PCI. Acute clinical management followed contemporary guidelines.

Cardiac MRI examinations were performed on a 3.0 T Philips Achieva TX system (Philips, Best, The Netherlands) equipped with a 32‐channel cardiac phased array receiver coil, MultiTransmit technology and high‐performance gradients with Gmax = 80mT/m and slew rate = 100 mT/m/msec.

### Scan Protocol

The protocol included a full LV contiguous stack of cine imaging and late gadolinium enhanced (LGE) imaging, three matching short‐axis slices (base, mid, and apex) for cDTI, T2 mapping and T2* mapping by acquiring the central three slices of five parallel short‐axis slices spaced equally from mitral annulus to LV apical cap.^10^

### MR Acquisition

Cine imaging was obtained using a balanced steady‐state free precession (bSSFP) pulse sequence (echo time [TE] 1.3 msece, repetition time [TR] 2.6 msec, flip angle 40°, and spatial resolution 1.6 × 2.0 × 10 mm). Modified Look‐Locker Imaging T1‐mapping parameters were as follows: 5/3/0 acquisition, TE 2.1 msec, TR 0.82 sec, flip angle 20°, spatial resolution 0.91 × 0.91 × 8 mm, SENSE 2 acceleration, cardiac delay time 777 msec, as described previously.^11^ T2‐ and T2*‐mapping was performed as previously described.^12^ In brief, a multiecho spin echo sequence, consisting of 24 refocused spin echoes subdivided into six consecutive groups, with turbo factor 4, and a linear k‐space order for each group, was used to map T2. Imaging parameters were acquisition matrix = 184 × 128, sensitivity encoding (SENSE) factor = 2, partial Fourier factor (in ky) = 0.6, TR/TE/echo spacing (msec) = 923/15/14, TSE shot duration = 90 msec, echo spacing between refocused echoes = 3.4 msec. T2* mapping was performed using multiecho gradient echo with similar imaging parameters as for T2 mapping, except for, readout of (Echoes) k‐space lines in each shot with each line for a separate image on the T2* decay curve (Echoes parameter), and this readout was repeated with different phase encodings (TFE factor) times every heartbeat (TFE factor) over (TFE shots) heartbeats (TFE shots). TR/TE1/echo spacing (msec) 15/2.3/2.2, black‐blood preparation pulse delay 420 msec, breath‐hold 15 seconds per slice, shot duration 148 msec, flip angle 20°, spatial resolution 1.8 × 1.8 × 8 mm. LGE imaging was performed at 10–15 minutes postcontrast (inversion recovery‐prepared T1‐weighted gradient echo, inversion time according to Look‐Locker scout, TR 3.7 msec, TE 2.0 msec, flip angle 25°, spatial resolution 1.75 × 1.75 × 8 mm).

### DTI Acquisition

DTI data were acquired using ECG‐gated second‐order motion‐compensated single‐shot spin echo planar imaging sequence^13^ with respiratory navigator slice tracking: (TE/TR = 89 msec/3RR intervals, flip angle = 90°, FOV = 238 × 238 mm, matrix size = 108 × 105, acquired in‐plane resolution = 2.20 × 2.27mm^2^, slice thickness = 8 mm, reconstructed voxel size = 1.7 × 1.7 × 8 mm^3^, 12 repetitions, sensitivity encoding [SENSE] acceleration = 1.8). Each cDTI dataset comprised 18 noncollinear diffusion‐weighted acquisitions with b‐values of 100 sec/mm^2^ (x3), 200 sec/mm^2^ (x3), and 500 sec/mm^2^ (x12). Based on cine data, trigger delay was set individually for each patient to coincide with approximately 60% peak systole and the center of k‐space was approximately at 85% of peak systole.

### Postprocessing

Cine, LGE and mapping sequences including T1, T2, and T2* were analyzed using cvi42 software (Circle Cardiovascular Imaging Inc, Calgary, Canada) to derive left ventricular volumes including end‐diastolic volume (LVEDV), left ventricular ejection fraction (LVEF) and tissue characteristics including infarct size, microvascular obstruction (MVO), native T1, T2, and T2* values. T2 and T2* maps were calculated using a maximum likelihood estimate fitting using the reconstruction software on the scanner.^14^ On LGE images, the threshold used for identifying infarcted tissue was set to 5 SD above remote myocardial tissue signal intensity.^15^ MVO was manually defined as dark zones within an area of LGE at 15 minutes postcontrast administration.

Raw cDTI data were postprocessed by an investigator (A.D., cardiologist with 3 years cardiac MR experience) blinded to clinical data, using custom‐built tools: these tools—based on MATLAB's (MathWorks, MA, USA) built‐in libraries—fulfilled DICOM image and metadata reading, interactive image rejection, image array operations including tensor fitting, and manual myocardial delineation. All diffusion‐weighted (DW) images were co‐registered via a mutual‐information‐based, multiresolution, affine scheme using the elastix toolbox.^16^ Quality control (QC) was undertaken by visual assessment by a second investigator (C.K., MR research fellow, 3 years cardiac MR experience) who was blinded from clinical data—this involved subjectively identifying DW images corrupted by artefact (unsuppressed fat, signal loss and visually appreciable suboptimal signal‐to‐noise ratio [SNR]) or failed registration and omitting them from further processing. After data rejection, 10 ± 2 diffusion‐weighted repetitions were available per diffusion gradient orientation for the construction of averaged DW images and tensor calculation. This was inclusive of base and mid slices only; apical data were excluded from the study due to persistent data quality issues. In cases where DW image rejection was inadequate for eliminating the effect of signal artifacts a further QC process was implemented whereby affected American Heart Association (AHA) segments were removed from quantification. Based on the registered data, magnitude images were averaged across accepted repetitions, according to diffusion direction and b‐value. Diffusion tensors were calculated using a linear least‐squares approach and used to derive MD and FA maps. Endo‐ and epicardial borders were manually delineated based on the reconstructed low diffusion‐weighted data (b = 100 sec/mm^2^).

Regions of interest (ROI) were manually planimetered for the analysis of MD, FA, native T1, T2, and T2* on corresponding maps. ROIs were manually drawn in accordance with standards set by the European Association for Cardiovascular Imaging.^17^ As recommended, ROIs were drawn on greyscale maps to avoid bias. Very small ROIs (<20 pixels) were avoided. ROIs were sampled away from endo‐ and epicardial borders to avoid the effects of partial voluming. In the acute scan, ROIs were drawn for each patient in areas of MVO if present (dark zone within LGE), infarct (positive for LGE, not inclusive of areas of MVO) and remote myocardium (opposite infarct). These ROIs were then used as a visual reference for sampling ROIs from the follow‐up scans, so that sampling occurred from near‐identical locations as the acute scan.

On T2* maps, an ROI with T2* < 20 msec was used to define the presence of iron.^18^ If iron was detected on T2* maps, the area was measured using manual planimetry, and copied across to the patient's cDTI, native T1 and T2 maps. On averaged DW images, the visual presence of an area of hypointense signal in the same region was defined as positive detection of iron. On native T1 and T2 maps, a relative reduction in relaxation times within this area compared to surrounding infarct myocardium constituted to a positive detection of iron. This visual analysis was undertaken by three separate observers to ensure agreement (A.D., C.K., and N.S.,—cardiologist with 2 years of cardiac MR experience). Further assessment of interobserver reproducibility is shown in the supplement (supplement Fig. S1).

### Statistical Analysis

Statistical analyses were performed in IBM SPSS Statistics 21.0 (Armonk, New York). Normality was checked using the Shapiro–Wilk test. Continuous variables are reported as mean ± SD. Comparison between quantitative variables was performed by independent‐sample parametric (unpaired Student's *t*‐test) or nonparametric (Mann–Whitney) statistical test as appropriate. For comparing results from initial and repeated measurements, paired *t*‐tests and ANOVA with Bonferroni post hoc comparisons were used. Comparison between categorical data was performed using χ^2^ tests. Using T2* mapping as the reference standard, the sensitivities of cDTI, T1, and T2 mapping for the detection of intramyocardial iron were assessed and the receiver operator characteristics (ROC) curves were plotted using the method of DeLong et al.^19^ The Bland–Altman method was used to assess the levels of agreement between cDTI and T2* mapping for estimating IMH size. All tests were assumed to be statistically significant when *P* < 0.05.

## Results

### Baseline Characteristics and Visit 1 MR

Demographics of the 50 patients who completed the acute scan at 5 ± 2 days are shown in Table 1. All patients underwent PPCI with a mean pain‐to‐balloon time of 246 ± 188 minutes and 45/50 patients (90%) had thrombolysis in myocardial infarction (TIMI) flow of 0 at the beginning of the procedure. TIMI 3 flow was restored in 48 of the 50 patients.

**TABLE 1:** Baseline Demographics

Demographics	All Patients (*n* = 50)	MVO at Acute Scan (*n* = 25)	No MVO at Acute Scan (*n* = 25)	*P* value
Age	60 ± 10	60 ± 11	60 ± 10	0.96
Male	40 (80)	22 (88)	18 (72)	0.94
BSA (m^2^)	1.9 ± 0.1	1.9 ± 0.2	2.0 ± 0.1	0.47
Current smoker	15 (30)	6 (24)	9 (36)	0.90
Diabetes mellitus	4 (8)	2 (8)	2 (8)	1.00
Hypertension	13 (26)	8 (32)	5 (20)	0.85
Family history	20 (40)	10 (40)	10 (40)	1.00
Infarct characteristics				
Pain to balloon time (minutes)	246 ± 188	252 ± 169	240 ± 207	0.86
TIMI flow pre‐PPCI	0.2 ± 0.7	0.0 ± 0.0	0.4 ± 0.9	0.03
TIMI flow post‐PPCI	3.0 ± 0.2	2.9 ± 0.3	3.0 ± 0.0	0.16
Culprit artery				
Left anterior descending artery	18 (36)	12 (48)	6 (24)	0.57
Left circumflex artery	9 (18)	5 (20)	4 (16)	0.99
Right coronary artery	22 (44)	8 (32)	14 (68)	0.65

Continuous variables are represented as mean ± SD and categorical variables are represented as *n* (%), PPCI, primary percutaneous coronary intervention; TIMI, thrombolysis in myocardial infarction.

**FIGURE 1:**
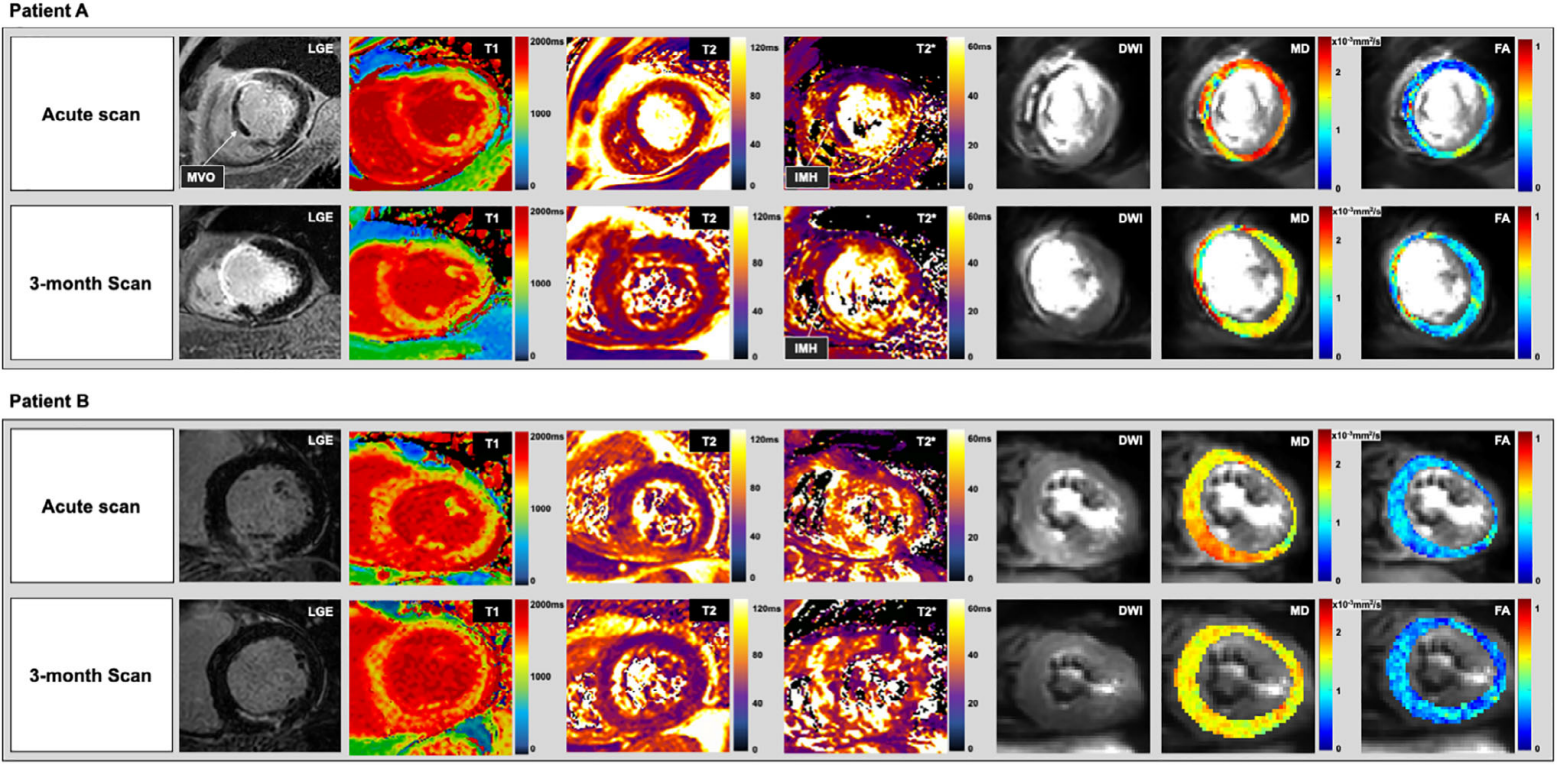
Patient A: Representative maps for a 63‐year‐old male who presented with anteroseptal ST‐elevation myocardial infarction (STEMI) and underwent primary percutaneous coronary intervention (PPCI) to his left anterior descending artery. In his acute scan, late gadolinium enhanced (LGE) image demonstrates extensive transmural infarction of the mid septal wall with microvascular obstruction (MVO). Native T1 and T2 relaxation times are increased in and around areas of infarction with a subtle area of hypointensity within the infarct. This hypointensity is considerably more visually evident on T2* and averaged diffusion weighted (DW) images. T2* within this area is <20 msec and is suggestive of the presence of iron from IMH. On MD mapping, values are relatively higher in and around infarction than remote, however within the areas of MVO, MD values are significantly lower. On FA maps, values show the opposite trend as MD. At the 3‐month scan for the same patient, LGE shows transmural enhancement of the septal walls. Native T1 and T2 relaxation times remain increased in areas of infarction, suggesting there is on‐going inflammation and edema. There is no longer any visual evidence of MVO, nor any area of low relaxation times on native T1 and T2 maps within the infarct; however on the T2* and averaged DW images, an area of hypointense signal within the infarct is still notable, indicating the presence of residual iron. MD remains decreased, and FA remains increased within these areas of hypointense signal, however in the surrounding myocardium, MD remains increased, and FA remains decreased in comparison to remote myocardium. Patient B: Representative maps of a 67‐year‐old male who presented with inferior STEMI and PPCI to his right coronary artery. In his acute scan, LGE image demonstrates hyperenhancement of the mid inferior wall with no evidence of MVO. Native T1 and T2 maps demonstrate hyperintense signals in and around areas of infarction, however in comparison to patient A, there is no area of hypointense signal on native T1, T2, T2* maps or averaged DW images. At 3 months, in the area of infarction, native T1 remains increased, however T2 relaxation times have decreased, signifying the resolution of oedema. There is still no area of hypointense signal on T2* and averaged DW images. MD has decreased in and around the areas of infarction, while there has been no notable serial change in FA.

CARDIAC MR results are displayed in Table 2. Mean acquisition time for cDTI was 13 ± 5 minutes. On the acute scan, LVEF was 43% ± 9% and the mean infarct size was 15 ± 11 g. MD was significantly higher in infarct zones compared to remote (MD_remote_ = 1.48 ± 0.07 × 10^−3^ mm^2^/sec, MD_infarct_ = 1.72 ± 0.14 × 10^−3^ mm^2^/sec and FA was significantly lower in infarct zones compared to remote (FA_remote_ = 0.36 ± 0.04, FA_infarct_ = 0.25 ± 0.04). Representative maps are shown in Fig. 1.

**TABLE 2:** Results From Acute and 3‐Month Scan

Cardiac MR Measurements	All Patients (*n* = 50)	MVO at Acute Scan (*n* = 25)	No MVO at Acute Scan (*n* = 25)	*P* value
LVEDV index (mL/m^2^)				
Acute scan	80 ± 19	83 ± 22	77 ± 17	0.17
3‐month scan	88 ± 24^a^	92 ± 30^a^	75 ± 14	0.03
LVEF (%)				
Acute scan	43 ± 9	37 ± 8	48 ± 6	<0.001
3‐month scan	48 ± 10^a^	42 ± 11^a^	54 ± 5	<0.001
Infarct mass (g)				
Acute scan	15 ± 11	21 ± 11	8 ± 6	<0.001
3‐month scan	10 ± 10^a^	15 ± 10^a^	5 ± 4	<0.001
MVO				
Mass at acute scan (g)	2.0 ± 4.0	3.8 ± 4.9	‐	‐
Persistent at 3‐month scan	0 (0)^a^	0 (0)^a^	‐	‐
Size at 3‐month scan (g)	‐	‐	‐	‐
T2* mapping				
Presence of IMH at acute scan	24 (24)	24 (96)	0 (0)	<0.001
T2* within IMH at acute scan (msec)	‐	11.7 ± 3.7	‐	‐
Area of IMH at acute scan (cm^2^)	‐	1.89 ± 0.96	‐	‐
Persistent at 3‐month scan	22 (46)^a^	22 (96)^a^	0 (0)	<0.001
Diffusion tensor imaging				
Acute scan				
Remote MD (×10^−3^ mm^2^/sec)	1.47 ± 0.08	1.50 ± 0.06	1.45 ± 0.08	0.03
Infarct MD (×10^−3^ mm^2^/sec)	1.72 ± 0.14	1.75 ± 0.16	1.69 ± 0.11	0.09
Remote FA	0.36 ± 0.04	0.35 ± 0.03	0.37 ± 0.04	0.11
Infarct FA	0.25 ± 0.04	0.23 ± 0.03	0.26 ± 0.04	<0.01
Hypointense signal within infarct	25 (50)	25 (100)	0 (0)	<0.001
Area of hypointense signal (cm^2^)	‐	2.0 ± 1.0	‐	‐
MD within hypointense signal (×10^−3^ mm^2^/sec)	‐	1.29 ± 0.20	‐	‐
FA within hypointense signal	‐	0.40 ± 0.07	‐	‐
3 month scan				
Remote MD at 3 months (×10^−3^ mm^2^/sec)	1.47 ± 0.07	1.47 ± 0.08^a^	1.46 ± 0.06	0.51
Infarct MD at 3 months (×10^−3^ mm^2^/sec)	1.81 ± 0.14	1.86 ± 0.14^a^	1.76 ± 0.13	0.02
Remote FA at 3 months	0.35 ± 0.03	0.34 ± 0.03 *	0.35 ± 0.03	0.60
Infarct FA at 3 months	0.23 ± 0.03	0.22 ± 0.03^a^	0.23 ± 0.04	0.23
Persistence of hypointensity	23 (48)	23 (100)^a^	‐	‐
Area of hypointense signal (cm^2^)	‐	1.0 ± 0.33	‐	‐
MD within hypointense signal (×10^−3^ mm^2^/sec)	‐	1.35 ± 0.14	‐	‐
FA within hypointense signal	‐	0.38 ± 0.06	‐	‐
Native T1 mapping				
Acute scan				
Remote T1 at acute scan (msec)	1207 ± 68	1232 ± 48	1178 ± 45	<0.001
Infarct T1 at acute scan (msec)	1482 ± 111	1486 ± 72	1476 ± 135	0.77
T1 within IMH at acute scan (msec)	‐	1253 ± 143	‐	‐
Cases with reduced T1 within IMH (*n*, %)	‐	8 (32%)	‐	‐
3 month scan				
Remote T1 at 3 months (msec)	1190 ± 87^a^	1231 ± 41^a^	1149 ± 101	<0.001
Infarct T1 at 3 months (msec)	1395 ± 92^a^	1439 ± 72^a^	1348 ± 89	<0.001
T1 within area of IMH at 3 months (msec)	‐	1348 ± 194^a^	‐	‐
Cases with reduced T1 within IMH (*n*, %)	‐	0 (0%)^a^	‐	‐
T2 Mapping				
Acute scan				
Remote T2 at acute scan (msec)	49 ± 7	48 ± 7	50 ± 6	0.60
Infarct T2 at acute scan (msec)	54 ± 9	57 ± 7	51 ± 6	0.11
T2 within IMH at acute scan (msec)	‐	48 ± 12	‐	‐
Cases with reduced T2 within IMH (*n*, %)	‐	16 (65%)	‐	‐
3 month scan				
Remote T2 at 3 months (msec)	49 ± 5^a^	49 ± 5^a^	48 ± 5	0.44
Infarct T2 at 3 months (msec)	53 ± 6^a^	55 ± 6^a^	48 ± 5	0.03
T2 within area of IMH at 3 months (msec)	‐	53 ± 8^a^	‐	‐
Cases with reduced T2 within IMH (*n*, %)	‐	3 (13%)^a^	‐	‐

Continuous variables are represented as mean ± SD and categorical variables are represented as *n* (%). For comparison, segmental MD and FA values of healthy volunteers as previously published are as follows: MD: 1.47 ± 0.08, FA: 0.38 ± 0.03.^20^ Departmental 3.0 T scanner reference range for native T1 is 1190 ± 50 msec.

^a^ Based on 23 patients who returned for 3‐month scan.

LVEDV = left ventricle end‐diastolic volume; LVEF = left ventricular ejection fraction; MVO = microvascular obstruction; IMH = intramyocardial hemorrhage; MD = mean diffusivity.

**FIGURE 2:**
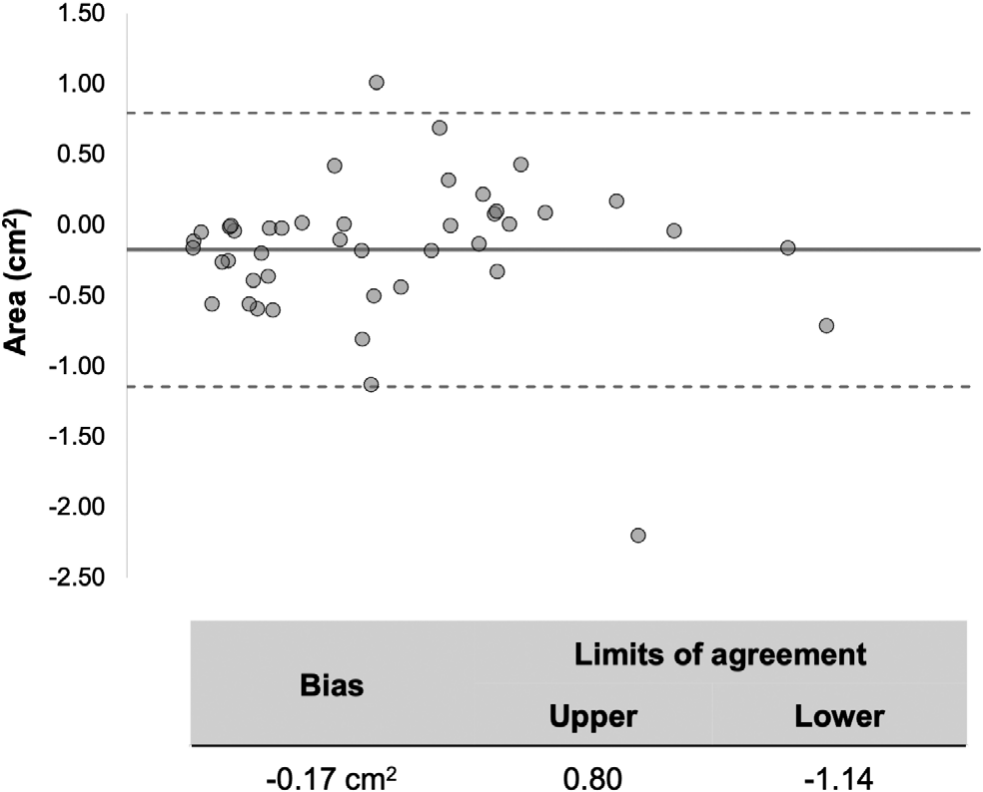
Comparison of IMH size estimation using T2* maps and cDTI. Bland–Altman plots demonstrate excellent agreement in the estimation of IMH size estimation using T2* and cardiac diffusion tensor imaging (cDTI), with a nonsignificant (*P* = 0.69) −0.17 cm^2^ bias using T2* mapping. The central (thick) line represents the bias and the dashed lines represent the 95% limits of agreement. There was a −0.17 cm^2^.

Twenty‐five patients (50%) had evidence of MVO on LGE images. In this subgroup, on T2* maps, 24 had evidence of IMH within their MVO (mean area 1.89 ± 0.96 cm^2^, mean T2* relaxation time 11.7 ± 3.7 msec). The one patient without IMH did have a hypointense region within the infarct on T2* maps, but the relaxation time of this area was 22 msec, just over the range for IMH. On averaged DW images, all 25 MVO patients had an area of hypointense signal within their MVO which matched the areas of IMH detected on T2* maps. The mean area of hypointense signal within infarct measured using averaged DW images was nonsignificantly greater than that detected by T2* mapping (2.0 ± 1.0 cm^2^ vs 1.89 ± 0.96 cm^2^, *P* = 0.69). Bland–Altman plots comparing the estimation of IMH size using cDTI and T2* mapping is shown in Fig. 2. In the remaining 25 patients with no MVO on LGE, 0 had evidence of IMH on T2* maps, 0 had areas of hypointense signal within their infarcted myocardium on averaged DW images, and 0 had areas of reduced relaxation times within their infarcts on native T1 and T2 maps.

### Differences Between Patients With and Without IMH on Acute Scan

In the acute scan, patients with IMH tended to have higher MD in their infarct myocardium surrounding the MVO than patients without MVO although the difference was not statistically significant (1.75 ± 0.16 vs 1.69 ± 0.11 × 10^−3^ mm^2^/sec, *P* = 0.09). In patients with IMH, FA was significantly lower in the infarct regions surrounding MVO than in infarct segments of patients without MVO (0.23 ± 0.03 vs 0.26 ± 0.04). In the acute scan, there was no significant difference in native T1 and T2 in infarct segments between patients with and without IMH (infarct T1_with IMH_ = 1486 ± 72 vs infarct T1_without IMH_ 1476 ± 135 msec, *P* = 0.77 and infarct T2_with IMH_ = 57 ± 7 vs infarct T1_without IMH_ 51 ± 6 msec, *P* = 0.11) (Table 2).

### Regional cDTI Changes Within Areas of IMH

**FIGURE 3:**
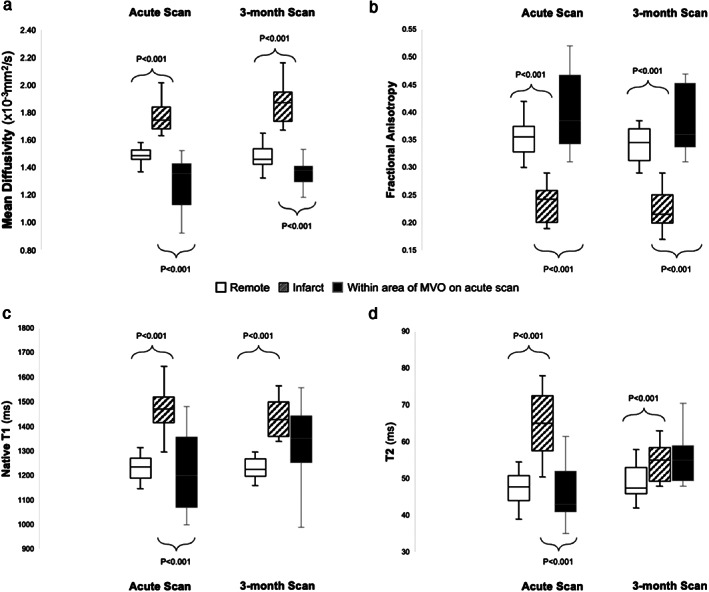
Regional variance in mean diffusivity (MD—panel a), fractional anisotropy (FA—panel b), native T1 (panel c), and T2 (panel d). In patients with microvascular obstruction (MVO) during the acute scan; MD, native T1 and T2 were all higher in the infarct myocardium than remote but significantly lower within the area of MVO compared to surrounding myocardium. By 3 months, this phenomenon persists with MD despite the visual absence of MVO. Meanwhile, FA shows the opposite trend; FA within MVO was significantly higher than surrounding infarcted myocardium in the acute scan, and this phenomenon also persisted at 3 months. On native T1 and T2 maps at 3 months, there is no longer a significant difference in relaxation times between infarcted myocardium and areas where there had been MVO on the acute scan.

With hypointense regions on averaged DW images, MD was significantly reduced in comparison with surrounding infarcted myocardium (1.29 ± 0.20 × 10^−3^ mm^2^/sec vs 1.75 ± 0.16 × 10^−3^ mm^2^/sec), while FA was significantly increased within these hypointense regions in comparison with surrounding infarcted myocardium (0.40 ± 0.07 vs 0.23 ± 0.03) as shown in Fig. 3.

### Follow‐Up MR at 3 Months

Forty‐eight patients returned for their follow‐up MR scan which took place at 104 ± 14 days. Two patients declined to participate in the follow‐up scan. At the 3‐month follow‐up, compared with acute scans, mean LVEF across the entire cohort had significantly improved to 48 ± 10%, and the mean infarct size had significantly reduced to 10 ± 10 g, across the entire cohort. Out of the 25 patients with IMH on the acute scan, 23 returned for their 3‐month scan. While none of the patients had visual evidence of MVO on LGE images; on averaged DW images, all 23 patients still had an area of hypointense signal within their infarct. T2* maps detected the presence of iron in 22 out of these 23 patients (96%) within these areas of hypointense signal. T2* had significantly increased between acute and 3‐month scan within these areas (T2*_acute_ = 11.7 ± 3.7 msec, T2*_3_ _months_ = 15.2 ± 3.8 msec), but there were no significant serial changes in infarct MD (MD_acute_ = 1.75 ± 0.16 × 10^−3^ mm^2^/sec, MD_3_ _months_ = 1.86 ± 0.14 × 10^−3^ mm^2^/sec, *P* = 0.08) or FA values (FA_acute_ = 0.23 ± 0.03, FA_3_ _months_ = 0.22 ± 0.03, *P* = 0.23) (Fig. 3).

### Differences Between Patients With and Without IMH at 3 Months

**FIGURE 4:**
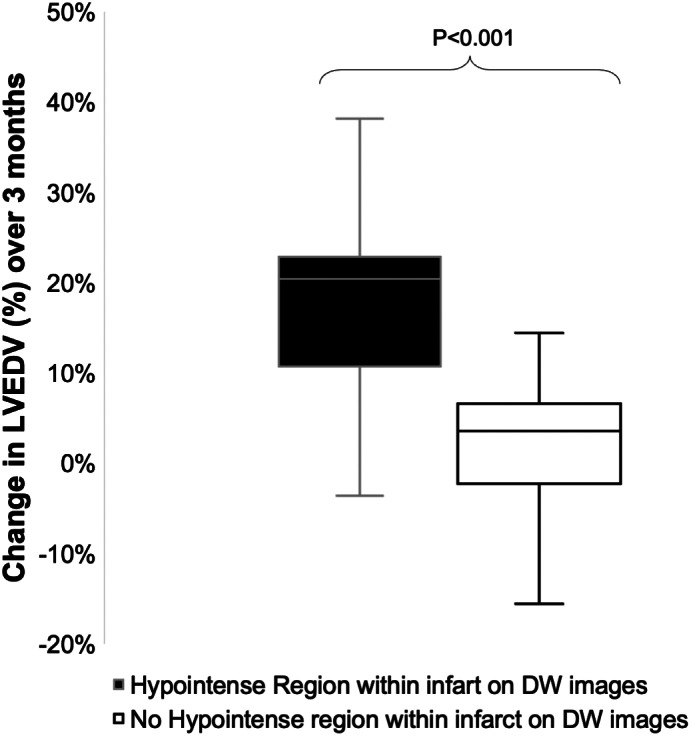
On averaged diffusion‐weighted (DW) images, patients who had an area of hypointense signal within their infarct experienced significantly greater increase in left ventricular end diastolic volume (LVEDV) over 3 months than patients who did not (19% vs 2%, *P* < 0.001).

Patients with persistently low T2* at 3 months had significantly higher MD, native T1 and T2 relaxation times in their infarct myocardium than patients without (MD 1.86 ± 0.14 vs 1.76 ± 0.13 × 10^−3^ mm^2^/sec, native T1: 1486 ± 72 msec vs 1348 ± 89 msec, T2: 55 ± 6 msec vs 48 ± 5 msec). In addition, patients with persistently low T2* also had significantly higher native T1 in their remote segments at 3 months than patients without persistent iron (1231 ± 41 msec vs 1140 ± 101 msec) Remote T2 at 3 months did not differ significantly between the two groups (IMH+ 49 ± 5 vs IMH− 48 ± 5, *P* = 0.44). Patients with hypointense signals within the infarct on averaged DW images on the acute scan experienced significantly greater increase in LVEDV by 3 months compared to patients without (19% vs 2%) (Fig. 4).

### Comparison of Sequences for the Detection of Iron

**FIGURE 5:**
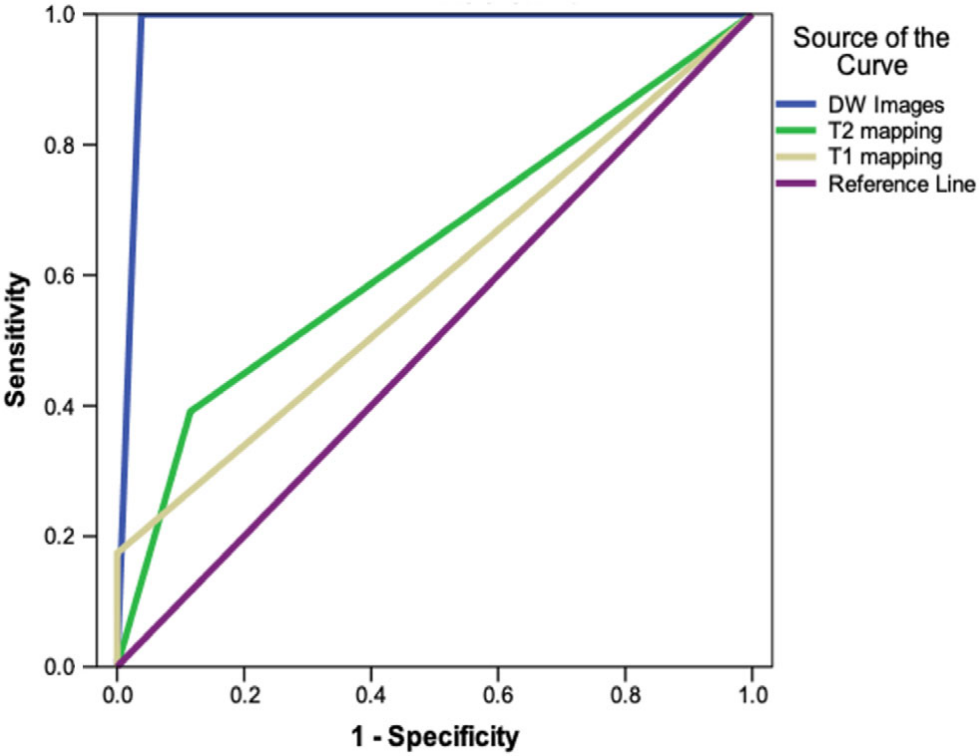
Receiver operator characteristics curves demonstrating the ability of averaged diffusion‐weighted (DW) images, T2 and native T1 maps to detect IMH as defined by T2*mapping, across 98 studies (50 acute and 48 3‐month scans). The blue line represents averaged DW images (area under curve [AUC] 0.981), the green line represents T2 maps (AUC 0.638), and the yellow line represents native T1 maps (AUC 0.587).

ROC curves for native T1, T2 maps and averaged DW images to detect IMH/residual iron as defined by T2* mapping is shown in Fig. 5. Across the 98 studies (50 acute, 48 at 3 months), DW images had higher area under curve (AUC) than native T1 (0.981 vs 0.587) and T2 mapping (0.981 vs 0.638) for the detection of IMH/residual iron.

### Impact of Reduced SNR on cDTI Parameters

In order to explore possible explanations for increased MD and reduced FA within areas of hemorrhage, simulation experiments were undertaken to assess the impact of reduced SNR on cDTI parameters. The results are shown in supplement Fig. S2.

## Discussion

Our results demonstrated that 1) compared with parametric mapping sequences, averaged DW images had superior sensitivity and specificity for the detection of IMH (as defined by T2*) on both acute and 3‐month cardiac MR scans post‐STEMI and 2) persistence of iron at 3 months detected by cDTI was associated with increased LVEDV, as well as increased T1 relaxation times in remote segments.

In the acute scans, MD and FA in remote myocardium matched previously reported values in healthy volunteers using spin echo DTI.^21^ All patients with evidence of IMH on T2* mapping had areas of hypointense signals within their infarct on averaged DW images. In the one subject with a “false positive” detection of IMH on averaged DW images, T2* was only marginally outside the category for IMH, indicating that there may have been a degree of hemorrhage but below the defined threshold. By 3 months, all patients with acute MVO had lost the visual appearance of MVO on LGE, however on averaged DW images, areas of hypointense signal within the infarct were seen in all of these patients. T2* relaxation times were significantly decreased in these areas (<20 msec) in most of the patients (96%), indicating the presence of residual iron. Therefore, our findings suggest that on averaged DW images, the presence of hypointense signal within chronic infarct segments is strongly suggestive of residual iron and is a remnant of severe ischemic injury in the past.

Previous studies have suggested that the deposition of crystalized iron in the myocardium carries a pro‐inflammatory burden; hence, myocardial segments with IMH can remain inflamed for up to 6 months, which in itself is associated with adverse long‐term adverse outcomes including LV remodeling.^5^, ^22^, ^23^, ^24^, ^25^, ^26^ In keeping with this, patients in our study with residual iron (defined using T2*) at 3 months also had significantly higher MD, native T1 and T2 relaxation times in their infarct regions than patients without residual iron, probably signifying on‐going inflammation.^27^ These patients also experienced a significantly greater increase in their LVEDV over the 3 months than patients without residual iron. Furthermore, they had significantly higher native T1 relaxation times in remote segments, which could signify diffuse interstitial remodeling.^28^ Therefore, the ability to detect the presence of iron at both an acute and chronic stage underlines a prognostically relevant, clinical utility of cDTI, although a greater duration of follow‐up is required to assess long‐term outcomes.

In keeping with findings from diffusion weighted imaging of the brain,^8^ our results also showed a decrease in estimated MD and an increase in estimated FA in areas of hemorrhage. The underlying mechanisms are thought to relate to the MR signal dephasing from magnetic inhomogeneities induced by local iron deposition. As a paramagnetic substance, hemosiderin causes variations in magnetic susceptibility on a microscopic scale, and this impacts the strength of the effective diffusion gradient. Interactions between the local gradients induced by iron oxide particles and the applied diffusion‐weighting gradients have the net effect of increasing the diffusion‐weighted signal, and reducing the apparent diffusivity.^29^ Furthermore, shorter T2 within the MVO reduces SNR and could result in an overestimation of the primary eigenvalue and an underestimation of tertiary eigenvalue due to eigenvalue repulsion..^30^, ^31^ As results from our simulation experiments show, this may contribute to an overestimation of FA but is unlikely to be the sole factor.

This finding has important implications; the influence of local iron deposition on diffusion measurements must be taken into consideration in the interpretation of diffusion data. When undertaking global and segmental analysis, the presence of iron can affect the overall average MD and FA. The use of ROI analysis approach can overcome this limitation, and we recommend this method for more accurate estimation of MD and FA in cases where iron may be present. Interpreting and quantifying the regional effect of iron on other DTI parameters such as helix angle and secondary eigenvectors is challenging. This is because unlike MD and FA, helix angle and secondary eigenvector values vary transmurally across the myocardium in healthy subjects. Due to this expected spatial inhomogeneity, it is challenging to define the “normal range” for a specific region within the myocardium, making ROI analysis unfeasible.

In conventional cardiac MR, spin echo sequences generally produce better image quality than gradient echo sequences. However, owing to the longer relaxation times required, gradient echo sequences are more sensitive to the presence of magnetic field inhomogeneities from iron, and hence are better suited for T2* mapping. In DTI, the application of diffusion encoding gradients makes spin echo more susceptible to the paramagnetic properties of iron due to the reasons discussed earlier, hence when comparing with native T1 and T2 mapping, DTI had better sensitivity and specificity for the detection of iron in our results. While previous studies have already demonstrated the capability of DTI to characterize myocardial microstructure,^6^, ^7^ something not possible with T2* imaging; results from this study highlight an additional clinical utility of DTI in detecting the presence of iron following STEMI, which is known to increase the risk of developing adverse remodeling and ventricular arrhythmia.^2^, ^5^

## Limitations

Recruiting patients following STEMI for complex acute and longitudinal imaging is challenging and the study sample size is therefore relatively small, but in keeping with similar studies.^2^, ^5^ Conclusions drawn from this study are based on correlations with published evidence and other cardiac MRI markers such as T2*, whereas validation with histological specimens would be preferable. The presence of susceptibility artefacts on T2* maps may confound appropriate detection of IMH. Historic T2* validation and thresholds were derived using 1.5 T scanners,^4^ while the present study was performed on a 3.0 T scanner. More recent comparative studies, however, have demonstrated that T2* on 3.0 T scanners show close association with 1.5 T scanners.^21^

## Conclusion

This study has shown that on DWI, the presence of hypointense signal within infarcted myocardium is suggestive of intramyocardial iron. Compared with native T1 and T2 maps, DWI provide superior contrast for the detection of intramyocardial iron; however, cDTI parameters such as MD and FA are affected by the paramagnetic susceptibility effects of iron; hence, caution is required in the interpretation of segmentally averaged cDTI data in segments where iron may be present.

## Conflict of Interest

The authors have reported that they have no relationships relevant to the contents of this paper to disclose.

## Supporting information


**Appendix S1**: Supporting Information
