# Supplementary material for: Detection of Intramyocardial Iron in Patients Following ST‐Elevation Myocardial Infarction Using Cardiac Diffusion Tensor Imaging
Source: J Magn Reson Imaging. 2022 Jan 12;56(4):1171–81. doi: 10.1002/jmri.28063 (PMC9544509; doi:10.1002/jmri.28063)
Supplement: Supplementary file 1 — Appendix S1: Supporting Information [file JMRI-56-1171-s001.docx]

**Supplementary Information**

**Interobserver reproducibility**

To assess interobserver reproducibility of IMH detection using cDTI maps, acute scans of 10 randomly selected patients with MVO were analysed by a separate investigator (**, MR research fellow with 3 years cardiac MR experience) who was blinded to the T2* maps. After planimetering areas of hypointensity on averaged DWI maps, the contours were copied across to MD and FA maps (as described in methods).

**Supplement Figure S1:** Bland-Altman plots comparing the area planimetered by ** and **, as well as the mean diffusivity (MD) and fractional anisotropy (FA) measurements from within the planimetered area. The central (thick) line represents the bias and the dashed lines represent the 95% limits of agreement. Overall, there was no significant bias in the estimation of IMH size, or MD / FA values between the 2 investigators.

**Simulation Experiments**

To assess the potential effects of SNR on the cDTI parameters, data in two example patients were further analysed by performing cDTI analysis, as described in Methods, based on subsets of data that include all 18 diffusion-weighting directions and b-values, and up to the first *n* repetitions, where 1 ≤ *n* ≤ 12 and *n* = 12 was the full dataset described in Results. Supplementary Figure 1 shows MD and FA in manually drawn ROIs in remote myocardium and MVO. The results show a positive bias in FA as the cumulative repetitions and hence SNR decreases, particularly at low *n*. At higher *n*, the bias in FA_n=6_ relative to FA_n=12_ averaged across both patients was +17% in remote myocardium and 8% in MVO. MD_n=6_ relative to MD_n=12_ was -2% and +2% in remote myocardium and MVO respectively. In both MD and FA, the heterogeneity across ROIs increased with lower *n*.

These results are consistent with previous reports describing SNR dependence in cDTI. [1–3] With further increases to SNR or cumulative repetitions beyond *n* = 12, further changes to MD and FA would be expected to decrease. We conclude that whilst SNR does influence DTI measurements, this does not account for the +74% higher FA seen in MVO compared to infarct regions.

**Supplementary Figure S2:** Mean diffusivity (MD) and fractional anisotropy (FA) reconstructed from subsets of data including the first 1 to 12 cumulative repetitions (mean SD ± across ROI). Data shown in two example patients, in slices where ROIs are available.

**Supplement Reference**

1. Jones DK, Basser PJ. “Squashing peanuts and smashing pumpkins”: How noise distorts diffusion-weighted MR data. Magn Reson Med. 2004 Nov;52(5):979–93.

2. Bastin ME, Armitage PA, Marshall I. A theoretical study of the effect of experimental noise on the measurement of anisotropy in diffusion imaging. Magn Reson Imaging. 1998 Sep;16(7):773–85.

3. Pierpaoli C, Basser PJ. Toward a quantitative assessment of diffusion anisotropy. Magn Reson Med. 1996 Dec;36(6):893–906.
